# Ultrahigh-mobility microbial cytochrome nanowires generate power from humidity

**DOI:** 10.21203/rs.3.rs-4724466/v1

**Published:** 2024-08-12

**Authors:** Peter Dahl, Jens Neu, Yangqi Gu, Catharine Shipps, Victor Batista, Nikhil Malvankar

## Abstract

Mixed electronic-ionic conductors are crucial for various technologies, including harvesting power from humidity in a durable, self-sustainable, manner unrestricted by location or environment ^1,2^ . Biological proteins have been proposed as mixed conductors for 50 years ^3,4^ . Recently, *Geobacter sulfurreducens* pili filaments have been claimed to act as nanowires to generate power ^5,6^ . Here, we show that the power is generated by *G. sulfurreducens* -produced cytochrome OmcZ nanowires that show 20,000-fold higher electron conductivity than pili ^7^ . Remarkably, nanowires show ultrahigh electron and proton mobility (>0.25 cm2/Vs), owing to directional charge migration through seamlessly-stacked hemes and a charged, hydrogen-bonding surface, respectively. AC impedance spectroscopy and DC conductivity measurements using four-probe van der Pauw and back-gated field-effect-transistor devices reveal that humidity increases carrier mobility by 30,000-fold. Cooling halves the activation energy, thereby accelerating charge transport. Electrochemical measurements identify the voltage and mobilities required to switch pure electronic conduction to mixed conduction for power generation. The high aspect ratio (1:1000) and hydrophilic nanowire surface captures moisture efficiently to reduce oxygen reversibly, generating large potentials (>0.5 V) necessary to sustain high power. Our studies establish a new class of biologically-synthesized, low-cost and high-performance mixed-conductors and identify key design principles for improving power output using highly-tunable electronic and protein structures.

Materials that simultaneously conduct ions and electrons are crucial for next-generation bioelectronic, optoelectronic, healthcare, and energy technologies, such as fuel cells, electrolyzers, and reactors[1]. They can also harvest energy from the humidity available anywhere on earth[5], thus offering the promise of clean, self-sustained power systems unrestricted by specific environmental requirements for solar or wind.

However, mixed conductors are challenging to synthesize due to contrasting requirements for the conduction of ions and electrons. A molecular-level understanding of coupled ion and electron dynamics is needed to co-design materials that efficiently conduct both carriers and have novel properties absent in a pure electronic or ionic conductor[2].

Biological proteins have been proposed as mixed conductors for 50 years but require very high (15 V) bias[3,4,8]. Deprotonating acidic amino acids in synthetic peptides improves the conduction of protons, but net conductivity remains low (σ ~ μS/cm), limiting their practical applications[9].

Surprisingly, living *G. sulfurreducens* biofilms show high ionic and electronic conductivities (σ ~ mS/cm), 1000-fold higher than synthetic peptides, due to hair-like surface appendages called nanowires[10-12] **(Fig. 1)**. Pili made up of PilA-N protein are hypothesized to serve as nanowires[5] in biofilms[6] for converting humidity into power[13]. However, this hypothesis is open to question because (1) *G. sulfurreducens* produces two types of filaments, one made up of cytochromes containing heme chain[7,14-17] **(Fig. 1c)** and second, pili composed of PilA-N and PilA-C proteins[7,15]. (2) The presence of the PilA-N monomer in filament preparations is taken as evidence for surface-displayed PilA-N filaments[18]. However, this method is prone to cellular contamination and detects intracellular PilA-N[7]. (3) PilA-N antibody binding to filaments is, in fact, imaged with filaments also containing PilA-C[19] or at a magnification where filaments are not identifiable[20]. (4) The 3 nm-diameter filaments claimed to be made up of PilA-N[21] are found to be DNA[7,15]. (5) Heterologous expression of *pilA*-N was purported to produce pili filaments without direct biochemical evidence[22]. (6) The conductivity of individual filaments produced heterologously is measured across the diameter under compression[23], not along the length. (7) Structural analysis of filaments claimed to be pili showed helical features consistent with cytochrome nanowires[17].

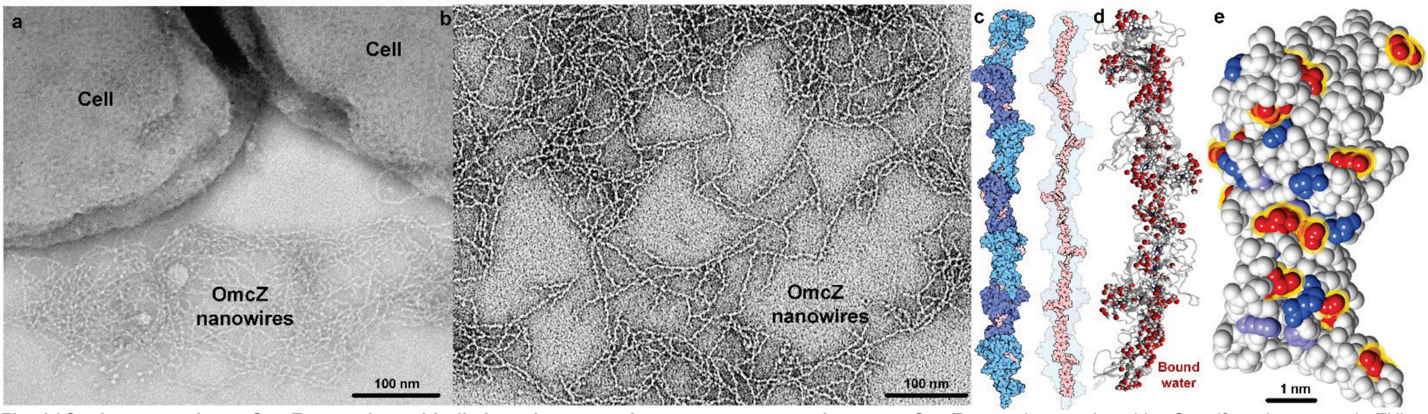

**Fig. 1 |*Geobacter* produces OmcZ nanowires with distinct electron and proton transport pathways. a,** OmcZ nanowires produced by *G. sulfurreducens* strain ZKI genetically engineered to overproduce OmcZ. **b,** OmcZ nanowires purified from cells. **c-e,** the structure of OmcZ nanowire showing **c,** hemes (pink) forming an electron transport channel and **d,** hydrogen bonds in bound water (red) and **e,** charged amino acids forming a proton transport channel. Acidic residues are in blue, and basic residues are in red with a yellow halo. (PDB ID: 7QL5)

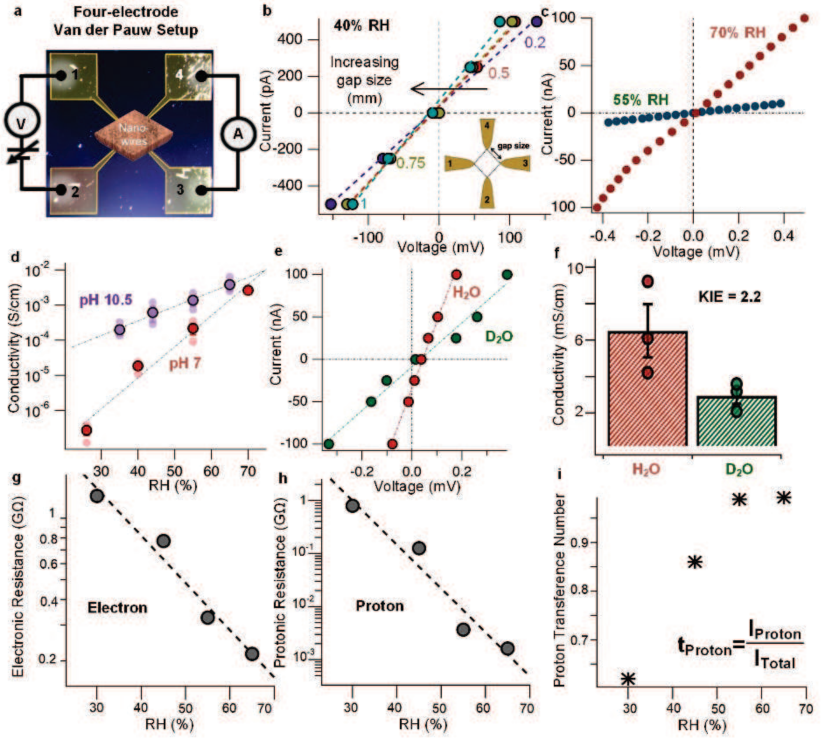

**Fig. 2 | Higher humidity increases nanowire conductivity 30,000-fold by increasing electronic and protonic currents. a,** Strategy to measure DC conductivity of nanowires using a 4-probe Van der Pauw setup. An optical image of the device shows distinct voltage and current probes. OmcZ nanowires were placed at the center. **b,** Validation of measurement approach. Similar nanowire conductance across 0.2 to 1 mm gap size confirms the uniformity of nanowire film. **Inset**, Schematic of device showing the identical gap size used across all probes. **c,** Representative current-voltage (I-V) curves of OmcZ nanowires at 55 % and 70 % relative humidity (RH) at pH 7. **d,** Higher humidity increases nanowire conductivity at pH 7 and 10.5. Replicates in light color and average in dark color. **e,** Representative I-V curves and **f,** corresponding conductivity values decreasing upon deuteration, thus demonstrating kinetic isotope effect (KIE). Error bars, s.e.m. (n=3). Higher humidity decreases both **g,** electronic, and **h,** and protonic resistance of nanowires measured using four-probe electrochemical impedance spectroscopy (EIS) spectra shown in Ext. Data Fig. 3. Two probe measurements also yielded similar results (Ext. Data Fig. 4). **I**, The transference number (t) shows the dominance of proton current in nanowires at higher humidity.

Thus, it is critical to determine the composition and electronic properties of nanowires produced by *G. sulfurreducens* biofilms to understand the power generation mechanism and improve the power output.

Of 111 cytochromes present in *G. sulfurreducens*, OmcZ is the only known nanowire-forming outer-surface cytochrome essential for the formation of high-power-density biofilms that require long-distance (>10 µm) electron conduction[24,25]. OmcZ-like nanowires are specifically overexpressed during power production[26] from methane-consuming archaea and their giant extrachromosomal elements called Borgs[27], further highlighting the importance of OmcZ. Pili are not extracellular in biofilms generating power[7] but are necessary for translocating OmcZ to the bacterial surface[7]. Therefore, we hypothesized that OmcZ nanowires generate power from humidity in G. sulfurreducens biofilms instead of pili.

The OmcZ nanowires are ideal for mixed conduction because individual nanowires show ultrahigh conductivity (>30 S/cm) due to closely stacked heme molecules forming a continuous path for electrons[24,25] (**Fig. 1c**). These nanowires also have the potential for high proton conductivity due to a chain of bound water forming an interconnected hydrogen-bonding network that can function as a proton wire (**Fig. 1d**). Nanowire also shows closely packed charged amino acids on the surface **(Fig. 1e).** Acidic residues glutamate and aspartate on the surface (**Fig. 1e**, red with yellow halo) deprotonate upon hydration and thus can donate protons to the proton conduction channel[9]. Amino acid residues with side chains containing amine groups can enhance proton transport upon protonation, thereby forming proton holes in the conduction channel[9].

**OmcZ nanowire conductivity increases 30,000-fold with humidity.**

To directly evaluate the hypothesis that OmcZ nanowire is an efficient mixed conductor, we obtained pure OmcZ nanowires without any contamination from other filaments using a protocol we had previously developed to obtain the atomic structure of nanowires[24]. Briefly, we constructed *ΔomcS* mutant of *G. sulfurreducens* CL-1, which forms highly conductive biofilms[28]. We then used sucrose gradient and size exclusion chromatography methods to obtain pure OmcZ nanowires[24]. Biochemical analysis[24] and transmission electron microscopy (TEM, **Fig. 1b)** confirmed that the only filaments in the sample are OmcZ nanowires.

We measured the electrical properties of nanowires using the van der Pauw electrode configuration because this contact geometry has been shown for protein samples to achieve equilibrium with the environment[3] **(Fig. 2a)**. Additionally, this 4-electrode geometry ensures contact-free intrinsic conductivity of proteins[29]. Commonly used contact geometry that sandwiches proteins between two electrodes causes severe artifacts in protein conductivity measurements[3]. These conductivity artifacts are due to only half the surface area available for water absorption in sandwich geometry compared to van der Pauw geometry, which necessitates long timescales (several hours) to achieve equilibrium water content in the protein. All measurements were performed in a triaxially-shielded anaerobic chamber under controlled humidity, and the equilibrium current was used to determine conductance as described previously[7,17,29].

Current-voltage measurements across various gap sizes yielded ohmic response with similar nanowire conductance, validating our measurement methodology **(Fig. 2b)**. Furthermore, all current-voltage responses were reversible without any hysteresis in contrast to prior measurements of low conductive proteins[30]. Increasing the humidity enhanced the conductance of OmcZ nanowires by more than 30,000-fold at pH 7 **(Fig. 2c-d)**.

Deprotonated nanowires at pH to 10.5 also showed humidity-dependent conductance change, albeit at a much smaller rate **(Fig. 2d)**. As raising the pH is expected to lower proton concentration, this weaker dependence of nanowire conductance on humidity at high pH suggested that the humidity-dependent conductance change is due to protons. Raising the pH can also alter the reduction potentials of hemes due to the protonation of heme propionate **(Extended Data Fig. 1).** However, the conductivity decrease due to raising the pH is small (< 10-fold)[25]. Therefore, we focused on the role of protons in humidity-dependent nanowire conductivity.

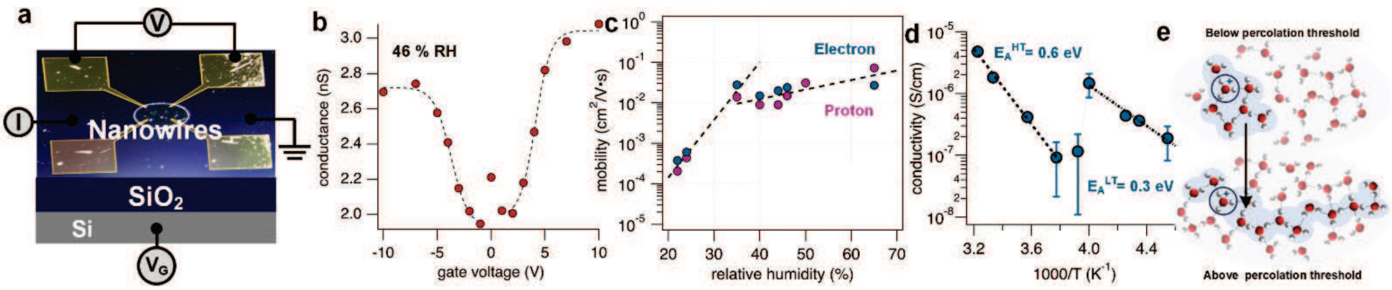

**Fig. 3 | A transition to a Grotthuss mechanism supports high proton mobility.** **a,** Schematic of the field-effect transistor device used to measure charge mobility. **b,** Sheet conductance of the OmcZ nanowire film, measured under varying gate voltage at 46 % RH (see Extended Data Fig. 6 for other RH). The black dashed lines are sigmoidal fits to the data. **c,** Humidity dependence of the mobility of electrons and protons extracted from our gating data. The black dashed lines are two separate exponential fits to the data. **d,** Temperature dependence of OmcZ film conductivity. The black dashed lines are exponential fits to the low temperature (LT) and high temperature (HT) regimes of the data. The corresponding activation energies are indicated on the graph. **e,** Representation of diffusion limited proton transport where the H-bond network is transformed from below percolation threshold to above percolation threshold for proton transport by a hopping mechanism, where a highly connected H-bond network supports exchange of the protons between water molecules, called Grotthuss mechanism.

Hydrating nanowires with $D_2O$ instead of $H_2O$ also lowered the conductance by 2.2-fold, exhibiting kinetic isotope effect[31] (KIE) **(Fig. 2e-f)**. The KIE further confirms the role of protons in nanowire conductivity.

To separate electronic and protonic current, we used electrochemical impedance spectroscopy (EIS, see methods for details)[10,11] **(Extended Data Fig. 2-4)**. Both electronic and protonic resistances decreased with increasing humidity, but proton current dominated the response, as revealed by the near unity (0.99) proton transference number (**Fig. 2g-i**). This response was specific to protons because other ions, such as lithium and sodium, did not impact ionic conductance (**Extended Data 5**), which is consistent with KIE results that are specific to protons. OmcZ nanowires thus show high electronic and protonic conductivity.

**OmcZ nanowires show ultrahigh electron and proton mobility.**

The mixed conductivity of OmcZ nanowires, indicative of ambipolar transport, suggested that the mobility of electrons and protons could be linked. High-performance mixed conductors require similar mobility for electrons and protons because the low-mobility carrier can limit the material performance[32,33]. To directly compare electron and proton mobility in OmcZ nanowires, we used back-gated field-effect transistor (FET) geometry in van der Pauw device configuration **(Fig. 3a. Extended Data Fig. 6)**. We measured the nanowire conductance for gate voltages ($V_G$) = -10 to 10 V. Back-gated FET response is rare in proteins due to extremely high electric fields required for the gating of molecular orbitals[34]. Surprisingly, OmcZ nanowires showed a gating response for both electrons and protons, evidenced by change in conductance for both positive and negative gate voltages **(Fig. 3b; Extended Data Fig. 5)**. We computed mobility using the relation $\mu = \frac{1}{C_i}\frac{dG}{dV_G}$, where $C_i$ is the specific capacitance of the oxide layer (1.12 F/m$^2$ for 300 nm $SiO_2$) and $G$, is the conductance of OmcZ nanowire film. Proteins typically show negligible charge mobility[35] but OmcZ nanowires showed exceptionally high mobility up to 0.1 cm$^2$/Vs. Notably, proton and electron mobility was comparable **(Fig. 3c).** Thus, OmcZ nanowires are ideal for high-performance mixed conductors by avoiding slow carriers that limit the efficiency[32,33] **(Fig. 3c)**.

**Humidity & cooling accelerate electrons and protons in nanowires.**

Mobilities increased exponentially with higher RH (**Fig. 3c),** but less rapidly at high RH. This is likely due to increased water content inducing a percolation transition from diffusion-limited to a proton-hopping (Grotthuss)[36] transport **(Fig. 3e)**. Below the percolation threshold, solvated protons exist in hydronium ion water clusters connected via a network of hydrogen bonds (H-bonds) where mobility is limited by thermally activated diffusion of the water cluster and exchange of H-bonds. Raising RH enhances H-bonds' connectivity above the threshold by increasing the water cluster's size, causing less rapid increase.

Similar to raising RH, cooling can also increase the connectivity of H-bonds by stabilizing the H-bonding network **(Fig. 3d)**. Upon cooling, the activation energy for the conduction along the nanowires halved (0.6 ± 0.02 eV to 0.32 ± 0.03). Similar decrease in the activation energy is a hallmark of high-performance conducting polymers that can also function as mixed conductors[1,37]. This facile conduction is consistent with rapid proton hopping between water molecules due to switching of proton transport from diffusion-limited to a Grotthuss mechanism[38,39] **(Fig. 3e)**.

**Both OmcZ and OmcS nanowires generate power from humidity.**

To evaluate whether the humidity-induced acceleration of electrons and protons in cytochrome nanowires contribute to power generation, we sandwiched a ~ 9 µm thick nanowire film between electrodes **(Fig. 4a, Extended Data Fig. 7)**. Without applying external voltage, we then measured the voltage drop ($V_{Drop}$) across a load resistor ($R_{Load}$ =220 KΩ) connected to the top and bottom electrode. We computed the power density ($P$) from the measured voltage drop using the relation $P=(V_{OCP} \times V_{Drop})/R_{Load} \times A$, where $V_{OCP}$ is the open circuit potential (OCP), and $A$ is the area of the bottom electrode (see methods for details). The power density increased exponentially with higher RH (**Fig. 4b-c),** similar to prior studies of *G. sulfurreducens* nanowires that claimed nanowires as pili[5].

OmcS nanowires also generated power from humidity, albeit 10 times lower than that from OmcZ, likely due to their lower electron conductivity **(Fig. 4c)**. The Aro5 strain purported to diminish pili conductivity was also able to generate power[5]. This was interpretated as electron transport is not not required for power generation[5]. However, the Aro5 strain produces OmcS nanowires[25], and thus, electron transport ability could also be important for power generation from *G. sulfurreducens* nanowires using coupled transport. As electrode-grown *G. sulfurreducens* used in prior work[5] produce OmcS and OmcZ nanowires and not pili[7], the power generation attributed to pili[5] could be due to cytochrome nanowires.

**A hydration gradient across nanowires causes power generation.**

To understand the power generation mechanism, we compared transport along the X-Y direction in thin OmcZ nanowire films (0.3 µm) to the transport in the Z direction in thick nanowire films (9 µm) used for power generation. We replaced the load resistor with a voltage source to apply an external bias (V) and measured current (I) through the nanowire film sandwiched between the top and bottom electrodes **(Fig. 4d).** I-V response in Z-direction was non-linear **(Fig. 4,e)** instead of linear observed in X-Y **(Fig. 2 b,c,e).** The non-linearity suggested an internal electrostatic gradient caused by an accumulation of positive charge toward the top of the device. This charge accumulation is likely due to an increased concentration of protons toward the top of the device where water content is maximal, thus creating a hydration gradient.

The slope of the I-V curve changed significantly around the OCP (x-intercept), and the OCP itself increased linearly with increasing RH **(Fig. 4e-f)**. As the non-linearity of the I-V curve is indicative of gradient and OCP reflects the magnitude of protons, these results suggest that the proton gradient increases at higher RH due to increased water content.

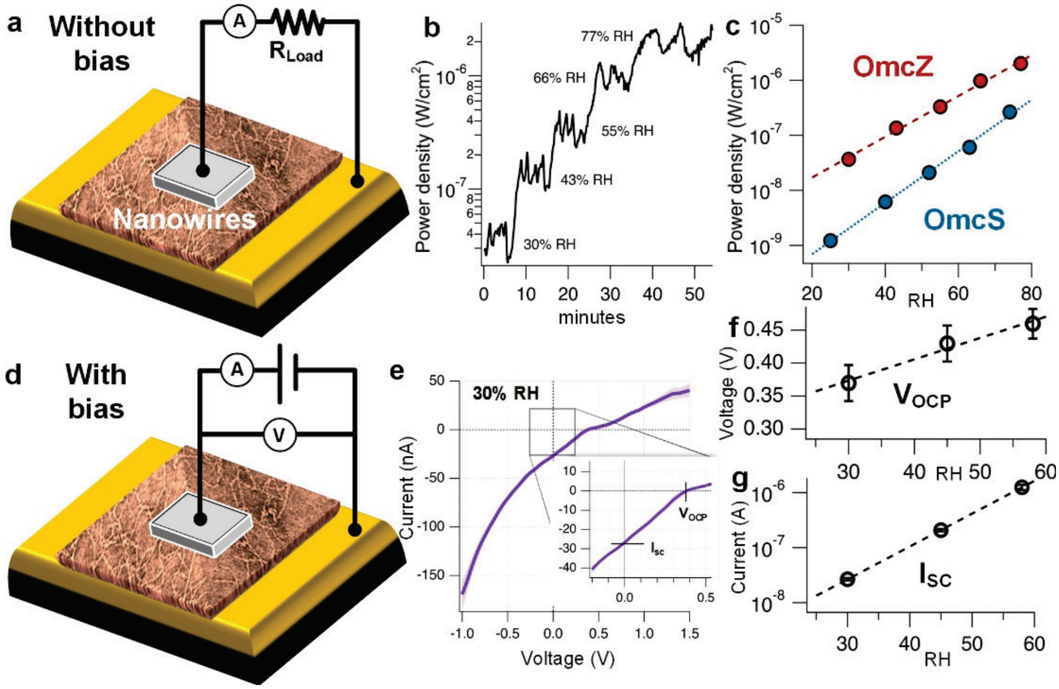

**Fig. 4 Cytochrome nanowires generate power from humidity.** **a,** Schematic of the power generation device. Nanowire films were sandwiched between top and bottom electrodes and voltage drop was measured across the load resistor. **b,** Power density generated by OmcZ nanowires increases with higher RH within minutes and is sustained for one hour. **c,** OmcZ nanowires show 10 times higher power density than OmcS nanowires. **d,** Schematic of the device used to measure current across nanowires under bias. Load resistor was replaced with a voltage source. **e,** Representative current-voltage curves across OmcZ nanowires with corresponding enlarged images at the location shown in a square. Increasing RH enhances **f,** open circuit potential ($V_{OCP}$) linearly and **g,** short-circuit current ($I_{SC}$) exponentially.

In contrast to the linear increase in OCP with RH, the short-circuit current ($I_{sc}$, y-intercept) increased exponentially with increased RH (Fig. 4f). Ion mobility determines the magnitude of $I_{sc}$. Therefore, the observed exponential increase in $I_{sc}$ is expected due to an exponential increase in proton mobility observed with higher RH **(Fig. 3c)**. Thus, proton mobility in nanowires dominates $I_{sc}$ generated from humidity.

To directly measure the proton mobility during power generation, we performed cyclic voltammetry by measuring the I-V in the Z-direction of OmcZ nanowire film **(Fig. 5a, Extended Data Fig. 8),** using the same electrode geometry used for power generation **(Fig. 4a,e)**. We observed a significant hysteresis between the anodic (increasing bias) and cathodic (decreasing bias) scans. This hysteresis indicates a substantial

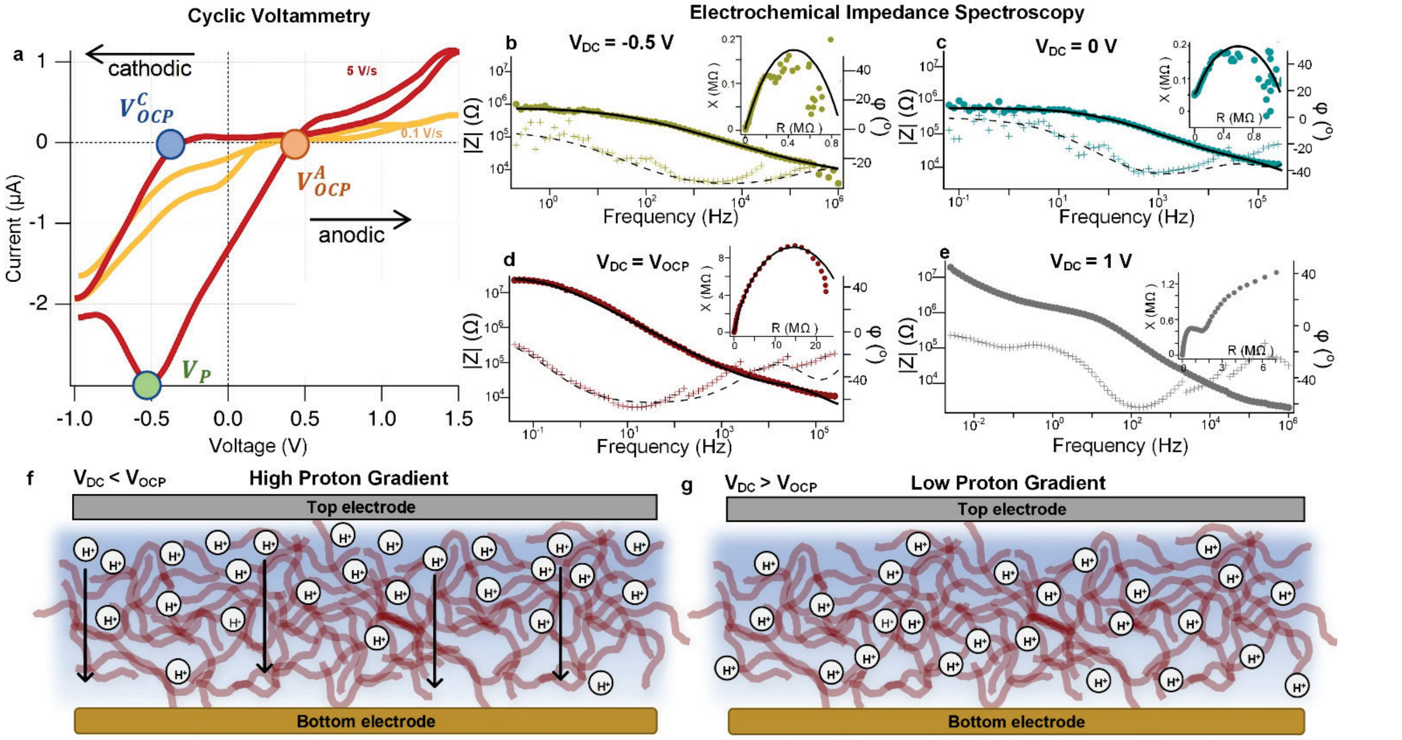

**Fig. 5 Coupled electron-proton transport confers ultrahigh mobility to nanowires to drive power generation from humidity. a,** Representative cyclic voltammetry of OmcZ nanowires using the device shown in Fig. 4e. Electrochemical impedance spectroscopy of OmcZ nanowires measured at $V_{DC}$ = **b,** -0.5 V, **c,** 0 V, **d,** $V_{OCP}$, and **e,** 1 V. plotted as Bode plots with magnitude (Z) and phase (φ) of the impedance along X and Y axes respectively. **Insets,** Nyquist plots with real (R) and imaginary (X) parts of impedance along X and Y axes respectively. **c,** OmcZ nanowires show 10-times higher power density than OmcS nanowires. Schematic of the re-equilibration of proton gradient by switching from **f,** high to **g,** low gradient as $V_{DC}$ becomes greater than $V_{OCP}$.

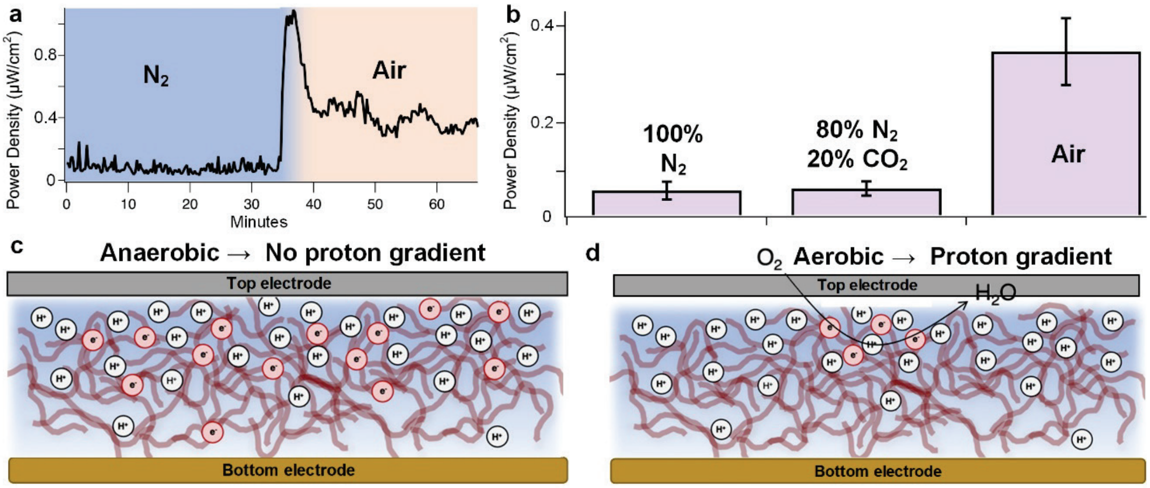

**Fig. 6 | Power generation is due to the proton gradient created by the oxidation of nanowire hemes. a,** Exposing OmcZ nanowires to air catalyzes power generation. **b,** Nanowires produce the highest power in air. Error bars s.e.m. (n=3). Schematic of nanowires showing **c,** no proton gradient due to buildup of electrons in reduced hemes neutralizing the protons and **d,** high proton gradient due to the loss of electrons in oxidized hemes, thus maintaining proton concentration.

rearrangement of internal charge gradients in the nanowire films. At higher scan rates (> 1V/s), we observed a prominent peak at -0.5V, but only in the anodic scan. To understand the origin of this peak, we compared the electron and proton conductivity at the peak voltage to other voltages using EIS measured at different DC offsets ($V_{DC}$). At $V_{DC} < V_{OCP}$, nanowires showed purely electronic conduction with 1 MΩ impedance, as indicated by a single semicircle in the Nyquist plot **(Fig. 5b-c).** At $V_{DC} = V_{OCP}$, nanowires still showed a primarily electronic response, but the impedance increased 10-fold to 10 MΩ. At $V_{DC} > V_{OCP}$ **(Fig. 5d)**, the impedance kept on increasing with the appearance of asecond semicircle in the Nyquist plot, indicative of significant protonic transport **(Fig. 5e).** This increase in the electronic resistance was accompanied by the decrease in protonic resistance. This inverse relation between electron and proton resistance suggests that mobile protons migrate against the electrostatic gradient to neutralize the negative charge of mobile electrons. This proton motive force drives power generation without any need for external bias. Our measurements showed that the protonic transport initiates at $V_{DC} > V_{OCP,}$ and decreases electronic resistance by neutralizing mobile electrons.

**Coupled electron-proton transport confers high nanowire mobility.**

The full cathodic scan was possible only at high scan rates, suggesting that at decreasing bias, the proton gradient re-equilibrates over time **(Fig. 5a)**. Thus, the peak in the anodic scan likely represents re-equilibration of the proton gradient with the peak position indicating the timescale of the re-equilibration. Using Fick's law and the values obtained through cyclic voltammetry, we computed that the proton mobility in nanowires along the z-direction necessary to re-equilibrate the gradient is $30 \pm 4 \times 10^{-6}$ cm$^2$/Vs (see methods for details). Notably, proton mobility in the Z-direction is 1000-fold lower than that measured along the nanowires in the X-Y direction **(Fig. 3c)**. Due to nanowire's high aspect ratio (1: 1000), they are likely to maintain the coupling of electrons and protons only along their length and not across their diameter. Thus, 1000-fold difference in conductivity between X-Y and Z suggests that coupling the electrons and protons is critical to achieving ultrahigh mobility in OmcZ nanowires.

**A proton gradient created by nanowire heme oxidation catalyzes power generation**

To determine how the proton gradient sustains power, we compared the power density of OmcZ nanowires in various environments. We found that nanowires produce 4 times higher power in air than under anoxic conditions such as N2 and $CO_2$ atmospheres **(Fig. 6a-b).** This behavior is consistent with our finding of proton gradient driving power generation. Nanowires are expected to build up electrons in reduced hemes in non-oxic conditions, which can neutralize the protons, preventing the formation of a power gradient **(Fig. 6c).** In contrast, air-oxidized hemes are expected to lose electrons and maintain the proton gradient. Thus, atmospheric oxygen is critical in generating the power from nanowires.

**Electron and proton conductivity determine the net power density.**

The reduction of oxygen by the c-type hemes of OmcZ nanowires likely proceeds via the 4-electron reaction in which 4 moles of electrons and 4 moles of protons are consumed per mole of O2 to produce one mole of water: $O_2 + 4e^- + 4H^+ \rightarrow 2H_2O$. Using the steady-state power output by OmcZ nanowires measured at 60 % RH corresponded to the flow of 457 nA of current through the load resistor. This corresponds to the consumption of 1.18 pmol of $O_2$ per second and 4.7 pmol of protons per second. Assuming all $O_2$ and $H^+$ adsorbed into the nanowire surface are consumed, we estimate oxygen and protons are replaced at 0.13 mmol/sec and 0.94 nmol/sec, respectively (see Methods for details). Therefore, in an aerobic atmosphere, regeneration of the OCP is limited by proton availability. The need to supply protons continuously could explain the decrease in power over time **(Fig. 6a)**.

Electron conductivity can significantly impact proton availability. Due to higher electron conductivity, nanowires can move electrons away from the cathode more rapidly, resulting in a larger effective surface area for the oxygen reduction reaction. Higher conductivity of OmcZ nanowires than OmcS nanowires could account for the 10-fold higher power density generated by OmcZ nanowires than OmcS nanowires.

**Discussion**

In summary, these studies establish that *G. sulfurreducens* grown on electrodes produce cytochrome nanowires to generate power from humidity and not pili as previously thought[13]. Identifying the materials responsible for power generation can improve power output by combining protein design using synthetic biology with device optimization. Cytochrome nanowires with ultrahigh electron and proton mobility are ideal for converting electrostatic and hydrokinetic energy associated with water into electricity due to their low cost, facile synthesis, highly hydrophilic surface, and very large aspect ratio (1:1000) for increased surface area.

OmcZ nanowires could have several advantages over current technologies because: (1) Nanowires can be produced synthetically at high yield and purity using *in vitro* assembly[24]. (2) Nanowire surface can be designed to increase their hydrophobicity to absorb moisture more efficiently. (3) Enhanced hydrogen bonding can improve proton transport by enhancing ion conductivity. (4) Nanowires can decrease the internal resistance of batteries and speed up oxygen reduction by increasing electron conductivity. Our identification of the mechanism of power generation could also help to improve power output by increasing cathode surface area by using mesh electrodes to improve oxygen access and by improving device architecture to enhance proton and electron transport.

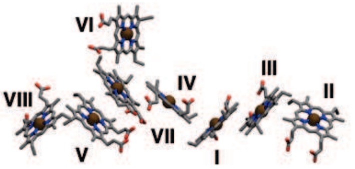

| heme | SASA (Å²) | $pK_a$ |
|---|---|---|
| I | 259 | 6.06 |
| II | 65 | 5.83 |
| III | 228 | 5.44 |
| IV | 61 | 7.58 |
| V | 175 | 8.22 |
| VI | 343 | 6.38 |
| VII | 89 | 11.91 |
| VIII | 88 | 6.45 |

**Extended Data Fig. 1 | Computed solvent-accessible surface area and pKa of the 8 hemes in the OmcZ protomer.** Solvent-accessible surface area (SASA) values were computed using the VMD program and pKa values were estimated using the PROPKA program.

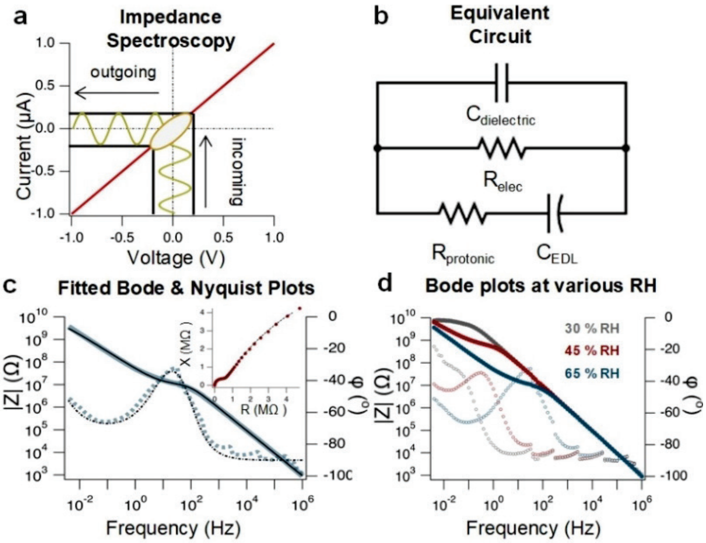

**Extended Data Fig. 2 | Electrochemical impedance spectroscopy (EIS) separates electronic and protonic currents. a,** Schematic of EIS. **b,** Equivalent circuit used to fit EIS data. **c,** Representative 2-probe Bode (cyan) and Nyquist (red) plots with fit (black). **d,** 2-probe Bode plots of OmcZ nanowires at various RH.

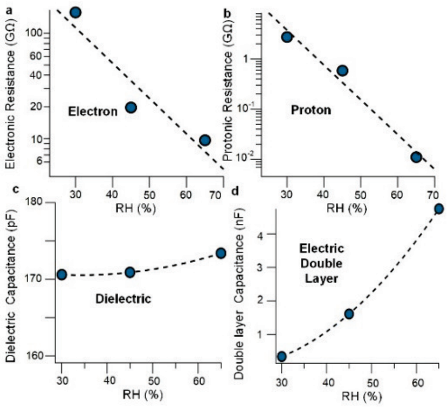

**Extended Data Fig. 3 | Equivalent circuit analysis of 2-probe and 4-probe EIS measurements of OmcZ nanowires. a,** Capacitance of the OmcZ film, extracted from the fit to the 2-probe impedance data for varying humidity. The black dashed line is an exponential fit to the data. **b,** Electric double layer capacitance extracted from the fit to the 2-probe impedance data for varying humidity. The black dashed line is an exponential fit to the data. **c,** Electronic resistance extracted from the fit to the 2-probe impedance data for varying humidity. The black line is an exponential fit to the data. **d,** Protonic resistance extracted from the fit to the 2-probe impedance data for varying humidity. The black line is an exponential fit to the data.

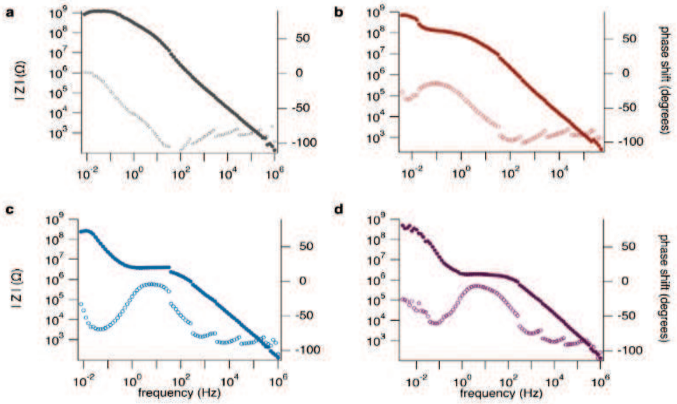

**Extended Data Fig. 4 | 4-probe EIS confirms the separation of electronic and protonic currents.** Bode plot for OmcZ nanowires measured in a 4-probe configuration on a van der Pauw electrode with 500 μm electrode spacing measured at following RH **a,** 30% **b,** 45% **c,** 55% and **d,** 65%,

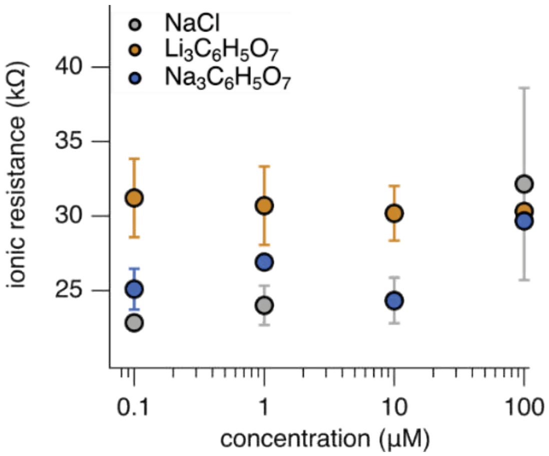

**Extended Data Fig. 5 | Confirmation of protonic nature of ionic conductivity in OmcZ nanowires.** Lack of change in ionic resistance of OmcZ films with added monovalent cations indicates that the change in ionic conductivity in OmcZ is due to protons and not ions in the buffer. Values represent mean ± s.e.m. (n≥2)

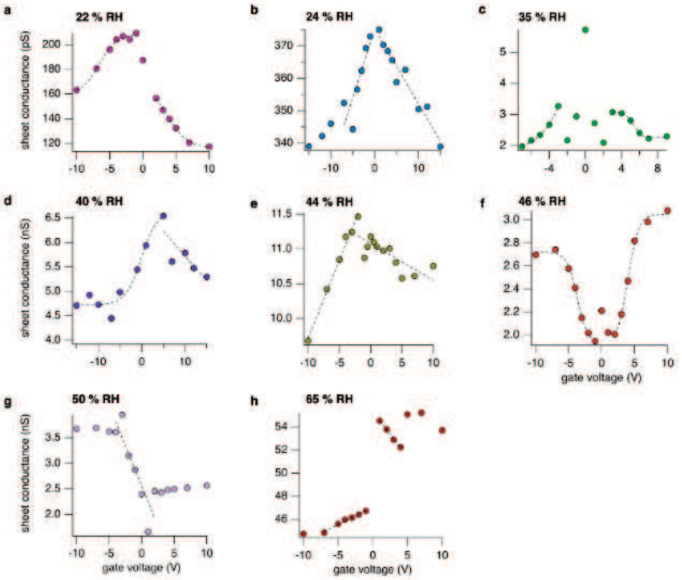

**Extended Data Fig. 6 | Field-effect back-gating measurements yielded overall reproducible behavior. a-h,** Sheet conductance of OmcZ nanowire films measured at gate voltages ranging from -10 to 10 V, labeled with RH used for measurements.

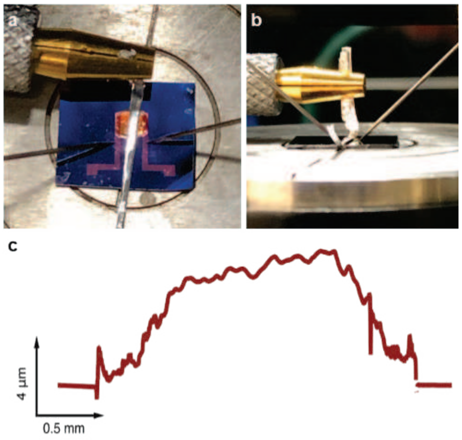

**Extended Data Fig. 7 | Hygroelectric power device. a,** top and **b,** side-view of hygroelectric power device with an OmcZ nanowire film sandwiched between bottom gold and top aluminum electrodes. **c,** Optical profile of an OmcZ nanowire film, used for thickness measurement.

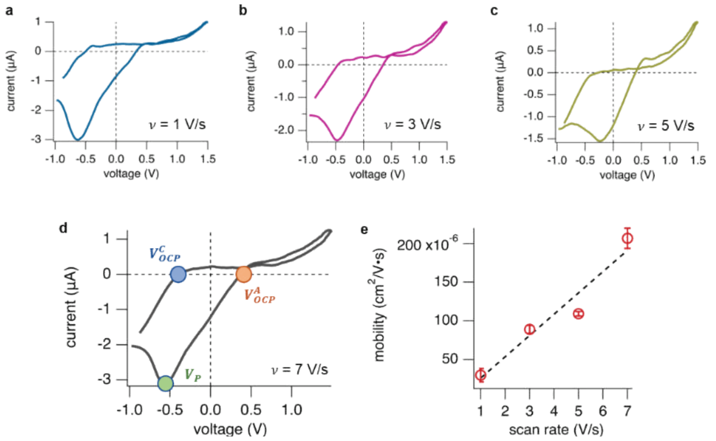

**Extended Data Fig. 8 | High scan-rate current voltage response. a-d,** Cyclic current-voltage response of OmcZ measured across the film thickness, measured at (a) 1 V/s, (b) 3 V/s, (c) 5 V/s, (d) 7 V/s. Panel (d) highlights the positions at which the three variables which are used to compute mobility are extracted. **e,** Mobility calculated from the current-voltage response at varying scan rates.

## Methods

**Bacterial growth conditions and nanowire preparation** To obtain pure OmcZ nanowires, we constructed *ΔomcS* mutant of *G. sulfurreducens* CL-1, which forms highly conductive biofilms[28] as described previously[24] or used ZKI genetically engineered to overproduce OmcZ[40]. Cells were grown in 10 L jugs at 25 °C to $OD_{600}$ ~ 0.8–1 in NBAF media and OmcZ nanowires were purified as described previously in a protocol used to solve the atomic structure of nanowires[24]. Briefly, cells were collected at ~15,000 × *g* for 12 min. Pellets were resuspended in 20 mM ethanolamine pH 10.5 and filaments were sheared from the cells using a blender for 1 min. The sample was run through a gel filtration column, Sephacryl S–500 HR, to obtain pure OmcZ nanowires. OmcS nanowires were purified from *ΔomcZ* mutant as described previously[41,42].

For transport measurements, 0.5 µl of the sample was dropped on the centre of the van der Pauw electrodes and allowed to dry overnight in a desiccator. Cytochrome nanowire hygroelectric devices were prepared as described. Briefly, a polydimethylsiloxane (PDMS) well with a radius of 2 mm was fixed to an IDA electrode. The electrode was placed on a hot plate at 60 °C. 12 µl of OmcZ (at 4 µM concentration) or OmcS (at 25 µM concentration), deposited in two 6 l drops in the PDMS well and allowed to dry.

**Negative staining transmission electron microscopy (TEM)** Electron microscopy science grids (mesh size 400) were plasma cleaned for 30 s in PDC-001-HP Harrick Plasma cleaner. About 5 µl of the sample (nanowires or cell culture) was dropped onto the carbon face of the grid and left to stand for 10 min. Excess solution was blotted. Samples were placed face down on 50 µL drops of negative stain (1% phosphotungstic acid, pH 6) for 30 s; the remaining solution was removed by blotting, then repeated. Grids were air-dried and stored in a sealed case until imaging using a JEM-1400Plus microscope operating at 80 kV (JEOL).

**Electrode design and fabrication:** This study used two types of electrodes. All transport measurements were performed using gold van der Pauw electrodes, while the hygroelectric power experiments used gold interdigitated array (IDA) electrodes. The IDA electrodes had spacings of either 3 µm or 5 µm. However, all connections were shorted together since these electrodes were used as a simple gold mesh-surface.

Both types of electrodes were prepared as described previously[41]. Briefly, the electrodes were fabricated using UV-Lithography on thermally oxidized Silicon wafers. This resulted in a 300 nm silicon dioxide layer, the insulation between the van der Pauw contacts and the dielectric for the back-gating experiments. The gold contacts were fabricated by spin coating a double resist, which was then exposed to UV radiation through a shadow mask and developed for 2 minutes. The photoresists were metalized using 5 nm Ti or Cr and 30-60 nm gold. Finally, a lift-off in 80-120° C NMP removed the metalized resists. A protective coating spin-coated onto the devices was washed off with acetone before use.

**Humidity variation** For the DC conductivity, EIS, charge mobility, and humidity dependence of power density measurements, relative humidity was varied by pumping humid air into a glovebox (Coy Lab) using compressed air. RH was monitored using a hygrometer (Fisherbrand).

For the anaerobic power generation measurement, a solution of saturated sodium chloride was sparged with $N_2$ gas for 3 hours before being moved into the glove box. Throughout the measurements, the solution was constantly bubbled with N2 or air, generating an environment with 60 % RH. The humidity was stable for the duration of the experiment.

**Electrical (DC) conductivity measurements** DC, electrical conductivity measurements, were performed in a four-probe, van der Pauw electrode geometry using a semiconductor Parameter Analyzer (Keithley 4200A-SCS), providing 0.1 fA current resolution and 0.5 µV voltage resolution. Measurements were conducted according to the van der Pauw method[43]. Briefly, the current was driven across one side of the device while the voltage difference between the two electrodes on the other side was measured. The voltage was allowed to settle over 1-3 minutes. Current-voltage curves were assembled with at least four points, including positive and negative currents. This was repeated for each of the four orientations of the electrode. sheet resistance was determined by solving the van der Pauw equation,

$$e^{\frac{-\pi R_{vertical}}{R_{sheet}}} + e^{\frac{-\pi R_{horizontal}}{R_{sheet}}} = 1 \qquad (1)$$

where, $R_{vertical}$ is the average of the resistance measured between contacts 1 and 2, and contacts 3 and 4 and $R_{horizontal}$ is the resistance measured between contacts 2 and 3 and contacts 1 and 4 (Fig. 2a). The van der Pauw equation was solved using the bisection method, implemented in a custom Python script. The sheet resistance was converted to conductivity using the equation

$$\sigma = \frac{1}{R_{sheet} d} \qquad (2)$$

where, d is the film thickness, measured using optical profilometry. For all DC conductivity measurements, at least 3 samples were measured.

For the kinetic isotope effect measurements, van der Pauw electrodes were placed on a sample puck and inserted into a sample wiring test station (Quantum Design). Electrical contact with the van der Pauw electrode was made by creating wire bonds between the electrode contacts and the sample puck using the operator-guided wedge-wedge wire bonded (West Bond), which uses ultrasonic energy to attach the aluminium wire to metal contacts. A custom, small humidity chamber was made from PDMS and fitted to the sample wiring test station. 1 ml of Milli-Q water (Millipore) or deuterium oxide (Cambridge Isotope Labs) was placed in the chamber. The sample was allowed to sit for 30 minutes to allow humidity to fill the chamber. Conductivity was measured by connecting the Parameter Analyzer to the sample wiring test station using triax-to-banana cable adapters. Due to small chamber size to fit hygrometer, it was not possible to measure the exact RH during the KIE measurements. Based on the magnitude of the conductivity measured at known RH, the estimated humidity was close to 75 % to 80 % RH.

**Charge mobility measurements** Field effect transistor devices were fabricated by scratching the back of the van der Pauw electrode wafers with a diamond scribe. Electrical connection to the n-doped silicon of the van der Pauw electrode wafer was achieved by adhering a plain gold electrode to the wafer using silver epoxy.

Measurements were performed using a semiconductor Parameter Analyzer (Keithley 4200A-SCS). The current drain was replaced with the ground unit to use all four preamplifiers available in the Parameter Analyzer. We measured a current-voltage curve using the ground unit as the current drain and compared it to a current-voltage curve measured with the current drain held at 0 V to ensure this would provide comparable results. The now free, fourth preamplifier channel was used to control the gate voltage. The gate current was monitored throughout the experiment to ensure the 300 nm SiO2 layer did not break down, or there was no significant leakage current.

We measured current-voltage curves at each gate voltage, providing 90 seconds for the voltage drop at each current value to settle. The sheet conductance of the device was determined as described under "Electrical (DC) Conductivity Measurements" and was plotted against gate voltage. The mobility was determined using the equation

$$\mu = \frac{1}{C_i}\frac{dG_S}{dV_G} \tag{3}$$

where, $C_i$ is the specific capacitance of the 300 nm $SiO_2$ oxide layer, $G_S$ is the sheet conductance, and $V_G$ is the gate voltage. The specific capacitance was calculated using the equation $C_i = {}^{\epsilon\epsilon_0}/_{d}$, where $\epsilon$ is the dielectric of $SiO_2$ ($\epsilon$ = 3.8), $\epsilon_0$ is the permittivity of free space ($\epsilon_0$ = 8.85 pF/m), and $d$ is the thickness of the oxide layer. The slope of the sheet conductance (${}^{dG_S}/_{dV_G}$) was determined by fitting a line to the steepest part of the sigmoidal dependence of the conductance on the gate voltage. This analysis was performed using Igor Pro software (WaveMetrics Inc.).

**Temperature variation conductivity measurements** As described previously[29,44], we used a Physical Property Measurement System (PPMS) (DynaCool, Quantum Design) to vary the temperature of a sample stage. Electrical contacts between the van der Pauw electrode and the sample stage were made by creating wire bonds between the electrodes and the pads of the sample stage using the operator-guided wedge-wedge wire bonder (West Bonder). After loading the sample to the PPMS, the chamber was purged three times, maintaining a minimal RH. All measurements were performed under ambient atmospheric pressure.

**Electrochemical impedance spectroscopy** (EIS). EIS and fast scan rate current-voltage curves in cyclic voltammetry (CV) were obtained with a Potentiostat (Gamry Instruments) as described previously[10-12] . For EIS, the frequency of the AC signal was scanned from 1 MHz to 5 mHz. For the two electrode measurements, the working and working source connections were connected to one electrode contact, and the reference and counter connections were connected to the other. For the four electrode measurements, the current source and current drain were connected to the working and counter connections, respectively. The working source and reference served as the voltmeter. The equivalent circuit to the data was fitted using the Gamry Echem Analyst™ software.

**Hygroelectric power experiments** Thick cytochrome nanowire films (>1 μm) were sandwiched between the gold IDA electrodes, upon which the films were deposited, and a 1 mm strip of aluminium foil. The open circuit potential was determined by obtaining a current-voltage curve using a semiconductor Parameter Analyzer (Keithley 4200A-SCS). The two electrodes were then attached to a 220 kΩ load resistor. We used the parameter analyzer to measure the voltage drop across the resistor. The power density was computed using the equation

$$Pd = \frac{V_{OCP}V_{drop}}{R_{load}A} \tag{4}$$

where, $V_{OCP}$ is the open circuit potential, $V_{drop}$ is the voltage drop across the load resistor, $R_{load}$ is the resistance of the load resistor, and $A$ is the area of the bottom electrode, coated by nanowires. The area of our IDA electrodes is 3 mm x 3 mm = 9 $mm^2$.

**Calculation of proton mobility from high scan rate current-voltage response** The current-voltage response of the thick OmcZ films in the hygroelectric devices, measured from top to bottom, demonstrated hysteresis when measured in cycles at high scan rates. Notably, the anodic (scanning from negative to positive) and cathodic (scanning from positive to negative) curves converged at a potential close to the open circuit potential of the device. The hysteresis was asymmetric around this point, with potentials less than the OCP, displaying a much more significant difference between anodic and cathodic current. Also, for sufficiently high scan rates (approx. ≥ 1 V/s), we observed a peak in the anodic scan at negative potentials.

EIS data collected at potentials greater than the OCP revealed a restructuring of the proton gradient (Fig. 5). Therefore, we attribute the increased hysteresis at potentials less than OCP to the migration of protons within the device. This is consistent with observing a negative shifted OCP for the cathodic scan (x-intercept). The peak in current in the anodic scan corresponds to the time the proton gradient equilibrates to its resting state, thus maximizing the internal driving force for electrons traversing the device.

Three data points identified within scans were used to extract the proton mobility. 1) The OCP of the device, measured in the anodic scan ($V_{OCP}^{A}$). 2) The OCP of the device, measured in the cathodic scan ($V_{OCP}^{C}$). 3) The potential at which we observe the peak in the anodic scan ($V_P$). We then used Fick's law to extract the proton mobility, as follows:

$$J = -D\frac{dc}{dx} \tag{5}$$

The concentration gradient which drives proton re-equilibration comes from the hydration gradient in the film. This can be estimated using $V_{OCP}^{A}$. We can use a Boltzmann distribution to determine the ratio of the number of protons in the top and bottom halves of the device:

$$r_{A(C)} = \frac{[H_{top}^{+}]}{[H_{bottom}^{+}]} = exp\left(\frac{V_{OCP}^{A}}{k_B T}\right) \tag{6}$$

The concentration gradient is computed as the difference between the number of protons in the bottom and top of the film divided by the dx, film thickness measured by optical profilometry (~9 μm) (Ext. Data Fig. 7c).

$$\frac{dc}{dx} = \frac{[H_{bottom}^{+}] - [H_{top}^{+}]}{dx} \tag{7}$$

The restructuring of the proton gradient is computed by plugging $V_{OCP}^{C}$ into Eq. (6). To compute the proton flux, requires the number of protons which migrate across the device to re-equilibrate the OCP, and the time required for this process to occur. The number of migrating protons is given by the difference in the number of moles at the bottom of the device obtained from the two OCP values. The re-equilibration time is given by

$$\tau = \frac{\left|\left(V_{lim}^{neg} - V_{OCP}^{C}\right) + \left(V_{lim}^{neg} - V_P\right)\right|}{\nu} \tag{8}$$

where, $V_{lim}^{neg}$ is the negative limit of the voltage scan (-1 V for our experiments) and $\nu$ is the scan rate.

Dividing the flux by the concentration gradient gives an expression for the diffusion coefficient, which depends only on the proton ratios computed from Eq. (6), the thickness of the film, and the re-equilibration time:

$$D = -\frac{(dx)^2}{\tau}\frac{r_A + r_C}{(1 - r_A)(1 + r_C)} \quad (9)$$

where $r_A$ and $r_C$ are the ratios of protons in the top and bottom of the film computed from Eq. (6) using $V_{OCP}^{A}$ and $V_{OCP}^{C}$, respectively. Finally, after plugging the flux and concentration gradient into Eq. (5), we can convert the diffusion coefficient (D) into proton mobility.

$$\mu = \frac{eD}{k_B T} \quad (10)$$

where e is the elementary charge, $k_B$ is the Boltzmann constant, and T is the temperature used for measurements (293 K).

This analysis can only be performed on data which shows a peak in the anodic scan. For the OmcZ nanowire films, we observed a peak for scan rates greater than 1 V/s. It is also essential to minimize the effect of capacitive current, which increases with increasing scan rate. Notably, we observe a linear increase in the computed mobility with scan rate (Extended Data Fig. 8e), which we attribute to capacitive effects. Capacitive contributions are minimal at a scan rate of 1 V/s, highlighted by the minimal hysteresis between anodic and cathodic scans for potentials greater than the OCP (Extended Data Fig. 8a).

**Calculating consumption rate for $O_2$ and $H^+$** To estimate the rate at which protons and atmospheric oxygen are replaced within the protein film, we used Eq. (5) to compute flux. At room temperature, the diffusion constant of gasses in air is approximately 0.2 $cm^2$/s. The concentration of $O_2$ is approximately 8.048 mM, and the concentration of protons (at 60 % RH) is approximately 60 nM. Assuming the concentration of $O_2$ and $H^+$ is zero at the bottom of the film, we calculate the concentration gradient by dividing the concentrations in the air by the thickness of the film. We multiply these values by the diffusion constant and obtain a flux. We multiply the flux by the area of the film to obtain the rate at which protons and $O_2$ are replaced.

**Reporting Summary** Further information on research design is available in the Reporting Summary.

**Data availability:** The paper includes all data sets presented in the study, as well as source data.

**Code availability** A custom Python script used in this work will be available on GitHub.

**Acknowledgements** This research was supported by the Human Frontier Science Program award no. RGP017/2023 (to N.S.M..), NSF CAREER award no. 1749662 (to N.S.M.), the NSF-ANR award no. 2210473 (to N.S.M.), and the National Institutes of Health (NIH) Director's New Innovator award (1DP2AI138259- 01 to N.S.M.).

**Author contributions** P.J.D. performed electrical and electrochemical measurements and analyzed data. J.N. fabricated devices. Y.G. and C.C.S. purified OmcZ nanowires and performed biochemical analyses. V.S.B. helped with data interpretation. N.S.M. supervised the project. and wrote the manuscript with P. J.D. with input from all authors.

**Competing interests** The authors declare no competing interests.
